# R-DEHM: CSI-Based Robust Duration Estimation of Human Motion with WiFi

**DOI:** 10.3390/s19061421

**Published:** 2019-03-22

**Authors:** Jijun Zhao, Lishuang Liu, Zhongcheng Wei, Chunhua Zhang, Wei Wang, Yongjian Fan

**Affiliations:** 1 School of Information & Electrical Engineering, Hebei University of Engineering, Handan 056038, Hebei, China; zhaojijun@hebeu.edu.cn (J.Z.); xindianyz@hebeu.edu.cn (L.L.); wangwei83@hebeu.edu.cn (W.W.); fanyj_ruc@ruc.edu.cn (Y.F.); 2 Hebei Key Laboratory of Security & Protection Information Sensing and Processing, Handan 056038, Hebei, China; 3 Department of Public Sports, Hebei University of Engineering, Handan 056038, Hebei, China; zhangchunhua@hebeu.edu.cn

**Keywords:** duration estimation, human motion detection, channel statement information, back propagation neural network, WiFi

## Abstract

As wireless sensing has developed, wireless behavior recognition has become a promising research area, in which human motion duration is one of the basic and significant parameters to measure human behavior. At present, however, there is no consideration of the duration estimation of human motion leveraging wireless signals. In this paper, we propose a novel system for robust duration estimation of human motion (R-DEHM) with WiFi in the area of interest. To achieve this, we first collect channel statement information (CSI) measurements on commodity WiFi devices and extract robust features from the CSI amplitude. Then, the back propagation neural network (BPNN) algorithm is introduced for detection by seeking a cutting line of the features for different states, i.e., moving human presence and absence. Instead of directly estimating the duration of human motion, we transform the complex and continuous duration estimation problem into a simple and discrete human motion detection by segmenting the CSI sequences. Furthermore, R-DEHM is implemented and evaluated in detail. The results of our experiments show that R-DEHM achieves the human motion detection and duration estimation with the average detection rate for human motion more than 94% and the average error rate for duration estimation less than 8%, respectively.

## 1. Introduction

With the rapid development of sensor networks, human behavior recognition is one of the research hotpots in various fields, such as smart homes, building surveillance, and medical health [1]. As a significant parameter of human behavior recognition, the duration of human motion has been researched widely by many researchers. There have been a lot of schemes for the duration of human motion in some scenarios of interest, including medical health [2], security monitoring [3], and even special places. Specifically, in medical health, the duration of postoperative rehabilitation training for patients affects the physical mechanisms of patients. In security monitoring, abnormal behavior of mobile personnel can be analyzed through duration. In special places, such as cold storage, chemical rooms with dangerous gases, and areas with strong radiation, the duration of human beings in the area of interest requires strict control. Therefore, the duration estimation of human movement in the scenario of interest is very important in research on human behavior recognition.

Previous works of human behavior recognition are vision-based [4,5], infrared-based [6], wearable-based [7,8], etc. However, there are some inherent limitations in traditional technologies. Vision-based approaches involve the privacy of target users and are significantly affected by light. Infrared-based methods are easy to block and fail to alarm. Furthermore, infrared devices need to be fixed in specific locations, resulting in poor mobility. Wearable devices, though flexible, need to be worn by objects, which makes users feel uncomfortable. Given the development of wireless communication, wireless devices are widely popular. There is an observation that wireless signals can be reflected by human bodies and present variance, which can be utilized to determine whether there are human beings in the area of interest or can even identify specific behavior. Therefore, considering the benefits of wireless signal detection, wireless human behavior recognition is increasingly becoming mainstream [1].

To make up for the limitations of traditional technologies, the received signal strength (RSS) of wireless communication from the medium access control (MAC) layer is ubiquitously utilized for human motion detection. There is an alteration for the RSS when the interest environment changes. Furthermore, it is adept at obtaining and lightening the burden of objects. Thus, researchers leverage existing wireless devices to capture the RSS from received packets and process it for human motion detection. Despite major advances, prior RSS based techniques are limiting with the following serious drawback: The RSS is highly variable to environmental changes, which can result in mistaken detection. Driven by the necessity of precise human behavior recognition systems, researchers have realized that a reliable metric is needed. Fortunately, channel state information (CSI) is obtained on commercial-off-the-shelf (COTS) devices through modified hardware by researchers to identify human behavior. Compared to the RSS, CSI has the following advantages: First, CSI maintains temporal stability in static environments and exhibits variance in a changing scenario; second, different from the RSS with spontaneous high susceptibility, CSI is independent of transmission power changes. Therefore, CSI has been touted as promising information for human behavior recognition. Nevertheless, there is a lack of research investigating the duration estimation of human motion using CSI.

In this paper, we propose a novel CSI-based robust duration estimation of human motion (R-DEHM) in the area of interest, considering multi-antennas with a simple majority-vote based detection algorithm. To achieve this, the human motion detection phase and duration estimation phase are included. We first collect CSI measurements on COTS devices and extract the features of different states of the scenario, i.e., static and dynamic. Next, a back propagation neural network (BPNN) algorithm is introduced to classify features for the detection of human beings in the area of interest. Finally, we apply a segmentation approach to estimate the duration of the moving human. Furthermore, to incorporate the detection results of all CSI streams for high accuracy, a majority-vote based algorithm is utilized in the human motion detection phase and duration estimation phase. R-DEHM is prototyped on the off-the-shelf Atheros AR9382 NIC. We evaluate the performance of R-DEHM in two real scenarios, including the working area and the living quarters. Experiment results demonstrate that R-DEHM achieves great performances on human motion detection and duration estimation.

In summary, the main contributions of our work are as follows:

- We transform the continuous and complex duration estimation problem into a discrete and simple human motion detection problem. Further, we present the design and implementation of R-DEHM in detail.
- A new metric is firstly introduced to evaluate the duration performance of human motion in wireless behavior recognition.
- We implement the system with off-the-shelf WiFi devices and evaluate its performance in two typical real environments. The experimental results demonstrate the effectiveness and robustness of the system.

In the remainder of this paper, the related work is reviewed in Section 2, and the preliminaries about R-DEHM are provided in Section 3. Section 4 introduces the methodology of our proposed scheme. Furthermore, the system performances are evaluated and discussed in Section 5. In Section 6, the overall work is summarized with conclusions.

## 2. Related Work

R-DEHM leverages wireless signals for human motion detection and duration estimation, which is closely related to two categories: RSS-based technology and CSI-based technology.

**RSS-based technology:** RSS, the MAC layer signature, has been prevalently applied to human motion detection due to its handy access to commodity devices. Specifically, the movement of personnel interferes with wireless signals and introduces variations of the RSS. Accordingly, moving humans can be detected by observing the fluctuation of the RSS. Leveraging this phenomenon, Jedari et al. [9] used the RSS based fingerprinting method to estimate the indoor location of a user or an object, which combined the k-nearest neighbor, a rules-based classifier, and random forest. Cassarà et al. [10] presented device free localization, utilizing wireless RSS in ambient assisted living scenarios. Kosba et al. [11] proposed a robust WLAN device-free passive motion detection system (RASID), which takes advantage of a statistical anomaly detection technique for human motion detection. RASID is robust in changing environments through building a non-parametric profile for signal strength readings. Soldovieri and Gennarelli [12] developed an improvised motion detection system (IMDS), exploiting a commercial smartphone as the access point and a laptop as the receiving terminal, to infer human presence by utilizing the variation of the RSS caused by shadowing and the multipath effect. The RSS achieved from commercial wireless devices can also be applied for gesture recognition. Pu et al. [13] and Abdelnasser et al. [14] presented gesture recognition systems by leveraging the minute Doppler shifts and different wireless signal change primitives, respectively. Although RSS-based human motion detection has been extensively researched, RSS-based technology cannot distinguish multiple signal propagation paths one by one since it measures the superposition effect of signal multipath propagation. Consequently, false detection often occurs in RSS-based detection systems, thus making it unfeasible.

**CSI-based technology:** The above limitation of RSS-based technology has motivated researchers to seek a new type of technology that optimizes the performance of human motion detection. Since commodity wireless network interface cards (NICs) in [15,16] were modified, a sample version can be obtained in the form of CSI on ordinary Wi-Fi devices. Compared to the conventional RSS, CSI provides finer-grained information with an amplitude and a phase [17]. Therefore, the fine-grained signature as a reliable metric handily replaces RSS to achieve more accurate device-free human motion detection. Wang et al. [18] presented an E-eyes scheme, which is an indoor device-free location-oriented activity identification system. The basic idea of E-eyes is to leverage the characteristics of CSI amplitude variations to classify activities in the area of interest. Zhu et al. [19] proposed a robust device-free through-the-wall detection of moving human (R-TTWD) scheme to leverage the penetrability of wireless signals. R-TTWD exploits the correlated CSI amplitude variations over different subcarriers and extracts first-order differences of the eigenvector of the CSI feature to implement through-the-wall (TTW) human detection. Alex et al. [1,20] proposed a channel state information based human activity recognition and monitoring system (CARM) that is a CSI-based activity recognition and monitoring system, building a CSI-speed model to characterize the relationship between CSI dynamics and human movement speeds. Furthermore, a CSI-activity model was built to portray the relationship between human movement speeds and human activities. CARM is implemented on COTS WiFi devices, leveraging CSI amplitude variation for activity recognition, and achieves an average accuracy of 96%. Gu et al. [21] designed a MoSense scheme, which is a radio frequency-based device-free motion detection system, exploiting ubiquitous WiFi signals. MoSense describes stationary states to distinguish motions with a silence analysis model and presents a distance-based mechanism to single out certain subcarriers for excellent motion detection. In addition to human motion detection and activity recognition, CSI-based technology is proverbially applied to fall detection [22,23], keystroke recognition [24,25], vital signs monitoring [26], counting [27,28], and so on. Commonly, the physical (PHY) layer signatures of CSI, characterizing the small-scale multi-path components, are more sensitive to human motion. In this paper, we explore a new CSI metric based on the PHY layer to detect and estimate the duration of dynamic human motion. To the best of our knowledge, this is the first work to apply fine-grained CSI to estimate the duration of human motion.

## 3. Preliminaries

This section introduces the core background of R-DEHM, including the PHY layer information and the MIMO technology.

### 3.1. PHY Layer Channel State Information (CSI)

As key information of the PHY layer, CSI is a measurement that characterizes the channel properties of a wireless communication link by combining the effects of time delay, amplitude attenuation, and phase shift [17]. Generally, a signal from the receiver is superposed since there are scatting, diffraction, and reflection phenomena in the signal channel propagation. The main purpose of CSI is to provide an adaption for the communication system within the current channel conditions. In this case, high reliability and high-speed communication can be guaranteed in the multi-antenna system.

In an orthogonal frequency division multiplexing (OFDM) system, the entire wireless channel is divided into multiple narrowband subcarriers. The CSI for subcarriers can be estimated by leveraging modern WiFi NICs. In our system, Atheros AR9382 is used for NICs with the maximum of N=114 subcarriers in the frequency domain, which is compatible with IEEE 802.11a/g/n/ac. Based on this, the PHY layer CSI can be estimated as shown in Equation (1):

(1) $$H(f)=[H(f_{1}),H(f_{2}),\cdots ,H(f_{k})],k\in [1,N],$$

where $H(f_{k})$ is the CSI at the subcarrier, k, with a centroid frequency, $f_{k}$. Further, each $H(f_{k})$ of the CSI portrays the amplitude and phase of the OFDM subcarrier, k, as shown in Equation (2):

(2) $$H(f_{k})=\left\|H(f_{k})\right\|e^{j\sin(∠H(f_{k}))},$$

where $\left\|H(f_{k})\right\|$ and $∠H(f_{k})$ represent the amplitude and phase of the centroid frequency, $f_{k}$, respectively.

For the sake of CSI extraction, two tools are currently available, i.e., Intel CSI Tool [15] and Atheros CSI Tool [16]. In this paper, a COTS router TL-WDR3500 is adopted as the transmitter to send data. Simultaneously, we have partiality for Atheros CSI Tool with Atheros NICs to implement R-DEHM.

### 3.2. Multiple-Input Multiple-Output (MIMO)

Multiple-input multiple-output (MIMO) technology leverages multiple antennas at the transmitter and receiver to enhance the quality of communication [29]. Based on the multiple antennas in MIMO, reliability can be increased via spatial diversity. Besides, the data throughput and the transmitting distance can be improved without enhancing the bandwidth and total transmitting power. Therefore, MIMO is considered a key technology in the wireless communication field.

In each antenna pair of the transmitter and receiver, the MIMO channel is composed of multiple subcarriers. The status of all MIMO channels is continuously monitored by wireless devices and characterized by CSI streams. Specifically, the CSI of all data streams can be portrayed as in Equation (3):

(3) $$H=\left[\begin{array}{cccc} H_{11} & H_{12} & \cdots & H_{1m}\\ H_{21} & H_{22} & \cdots & H_{2m}\\ \vdots & \vdots & \ddots & \vdots \\ H_{n1} & H_{n2} & \cdots & H_{nm} \end{array}\right],$$

where m and n represent the number of transmitter antennas and receiver antennas, respectively. In addition, $H_{nm}$ is a vector describing the CSI of all subcarriers between the *m*th transmitter antenna to the *n*th receiver antenna, and hence the total number of CSI streams is m×n.

## 4. System Overview

**Figure 1:**
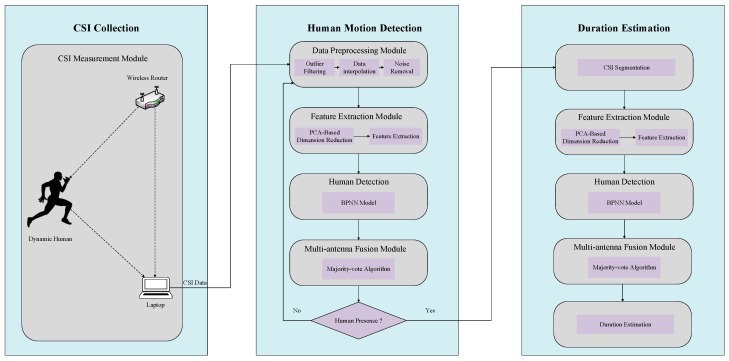
System architecture.

In this section, we present an overview of the R-DEHM architecture as shown in Figure 1. Two kinds of data streams exist, i.e., training data streams and testing data streams, collected with COTS devices. The difference between the two kinds of data streams is that the training data streams needs to be labeled artificially. The detailed procedure of R-DEHM is as follows:

- Firstly, the original training data needs to be preprocessed through the data preprocessing module since the raw CSI measurements could contain biased observations and noise. In our system, a Hampel identifier [30] is used for outlier filtering, 1-D linear interpolation for supplementing the information, and wavelet-based noise removal for denoising.
- Secondly, the feature extraction module is introduced to extract the feature of the filtered training data. In this module, we use principal component analysis (PCA) based technology to reduce CSI dimensions. Based on the correlation among different subcarriers, we further extract the ratio of the variance and the mean of the first-order difference to achieve a robust feature profile.
- Afterwards, a training model in R-DEHM is obtained by training robust features based on BPNN. For the sake of reliability, we incorporate a majority-vote algorithm in the results of all streams in the training model, considering multi-antenna.
- Then, to achieve the robust feature profile, a data preprocessing module and a feature extraction module for each testing data stream are also employed. Subsequently, we leverage the training model to confirm the statement of the presence or absence of each testing data. Besides, the majority-vote algorithm is also utilized in the multi-antenna fusion module to enhance the accuracy of human motion detection in our system.
- Finally, if a CSI data is confirmed as the statement of a presence in the scenario, the filtered data of the CSI, after the data preprocessing module, is divided into lots of segments with the same window size. Further, each segment undergoes feature extraction and the BPNN for human motion detection, and the multi-antenna fusion module is applied to ensure accuracy. According to the window size, the final results of all segments are integrated to estimate the duration of human motion in the scenario.

## 5. Methodology

In this section, we elaborate the design of R-DEHM with real measurements.

### 5.1. Data Preprocessing Module

Due to the noise in the CSI, the data preprocessing module is set for the raw signal, including outlier filtering, data interpolation, and noise removal.

#### 5.1.1. Outlier Filtering

**Figure 2:**
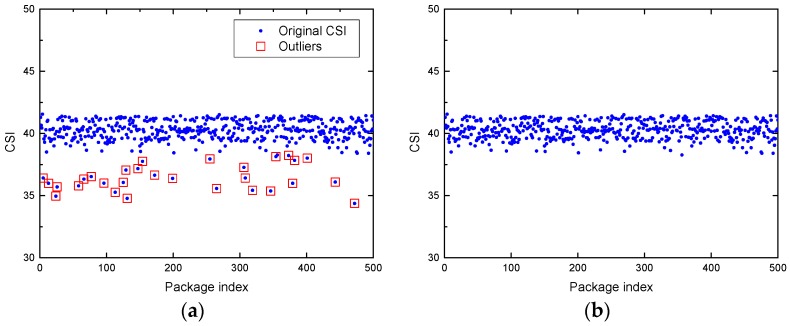
Outlier filtering and data interpolation for CSI measurement in the absence scenario: (**a**) The original CSI data of subcarrier 1 with outliers; (**b**) the CSI data of subcarrier 1 after outlier filtering and linear interpolation.

In the data preprocessing module, filtering outliers is the first procedure to dispose the original CSI measurement. In the middle of the experiment, due to the protocol specifications between the transmitter and the receiver, as well as the exotic environmental interference, there are many abnormal values that are apparently not triggered by human motion. Therefore, it is necessary to sift out outliers that affect the performance of human motion detection and duration estimation. To identify and filter out the outliers in R-DEHM, we exploit a Hampel identifier [30] to discriminate between cancerous data and normal data. The Hampel filter identifies the location of outliers that fall outside of the closed interval [μ−γσ,μ+γσ], where μ and σ represent the median and the median absolute deviation (MAD) of the data sequence, respectively, and γ is a parameter that is dependent on the applications. In our experiment, γ is adopted with the most extensive value of 3 [19]. Figure 2a illustrates the outlier observations, and it can be seen clearly that there are some abrupt changes observed from the overall trend, which are marked by red boxes.

#### 5.1.2. Data Interpolation

It must be noted that some locations with null CSI data exist after filtering outliers. In addition, no matter what the sampling rate is, sampling jitter is quite common in CSI, which results in CSI data packets being lost to varying degrees. Thus, to account for the CSI data loss caused by sampling jitter and outlier filtering, the CSI measurements must be interpolated. Specifically, the simplest 1-D linear interpolation algorithm is utilized in R-DEHM to ensure the integrity of the CSI measurements with 1 ms between consecutive measurements. Figure 2b presents the CSI measurements after performing outlier filtering and data interpolation effectively.

#### 5.1.3. Noise Removal

There is much noise in CSI measurement due to the hardware imperfections of the transceiver and the electromagnetic interference in complex indoor environments, such as surrounding electromagnetic interference, and air pressure and temperature changes. Hence, the measured CSI data are noisy after outlier filtering and data interpolation. As such, the interference is bound to conceal the distinctness of the statement features in the scenario. Therefore, it is of great significance to remove the noise of the measured CSI data after outlier filtering and data interpolation. Considering the variations caused by human activities with a low frequency range, a low-pass filter is adopted in R-DEHM to eliminate noise at the high end of the spectrum. According to the experiments, we argue that the conventional filter, e.g., moving average filtering, is not appropriate for denoising in R-DEHM, since it is unable to achieve the removal of random noises. However, wavelet analysis can simultaneously analyze a signal in both time and frequency domains. Moreover, it has the ability of multi-resolution analysis. Therefore, at different decomposition levels, wavelet analysis can effectively distinguish sudden changes of the signal and noise signal to implement noise removal. Ultimately, a wavelet-based denoising scheme is employed to eliminate messy noise and smooth the CSI measurement.

**Figure 3:**
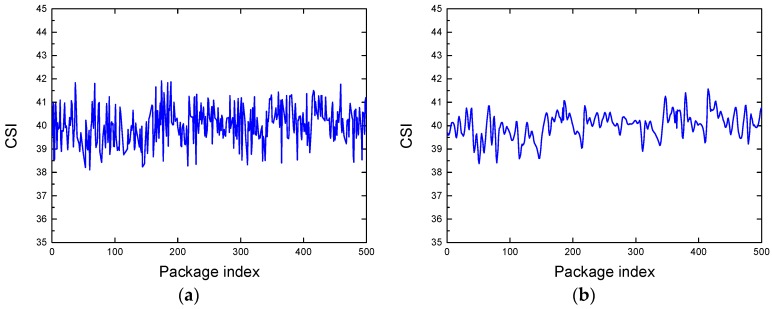
Noise removal for the CSI measurement in the presence scenario: (**a**) The raw CSI measurement; (**b**) the CSI measurement after removal with the wavelet-based denoising.

Wavelet analysis is the local analysis of spatial frequency, which consists of three stages: Decomposition, thresholding detail coefficients, and reconstruction. It progressively refines the signal by scaling and translation operation, thus achieving the time subdivision at high frequency and the frequency subdivision at low frequency. Specifically, the original CSI measurement is firstly decomposed by the wavelet to obtain high-frequency detailed components and low-frequency approximate components. Then, the threshold processing is applied to high-frequency detailed components for noise removal. Next, the processed components are reconstructed by the wavelet to obtain the denoised CSI measurement. Figure 3b shows the CSI measurement after using wavelet-based denoising when applied to the CSI measurement in Figure 3a in the presence scenario. It can be observed that the noise signal is almost removed from the raw CSI measurement.

### 5.2. Feature Extraction Module

In this subsection, we extract the feature for human motion detection. Firstly, the PCA-based dimension reduction is used to decrease the computational complexity of high dimensional CSI measurements. Then, we analyze the eigenvectors and corresponding principal components of the CSI measurements in order to extract the robustness features.

#### 5.2.1. PCA-Based Dimension Reduction

It is universally known that PCA transforms the original data into a set of linearly independent representations of each dimension by linear transformation. It can be used to extract the main feature components of the data, basically holding back all useful information of the previous data. Given the computational complexity of high dimensional CSI measurements with a maximum of N=114 subcarriers, we apply a novel PCA-based technology for the denoised CSI streams to reduce data dimensions, and to shorten the computational time of the system. Moreover, PCA is utilized to identify the correlations between adjacent subcarriers and unveil the most common variations among different subcarriers. In R-DEHM, PCA-based technology on each primary denoised CSI stream has the following four steps.

Firstly, let $H_{tc,tr}(i)$ be a N×1 dimension vector containing the CSI values of the N=114 subcarriers between an arbitrary antenna pair, tc−tr, for the *i*th CSI packet. Meanwhile, let $H_{tc,tr}$ be a S×N dimension matrix containing the CSI values of the N subcarriers between an arbitrary antenna pair, tc−tr, for S consecutive CSI packets. Specifically, $H_{tc,tr}$ can be characterized as shown in Equation (4):

(4) $$H_{tc,tr}={\left[H_{tr,tr}(1),H_{tc,tr}(2),\cdots H_{tc,tr}(s)\right]}^{T},$$

where the columns of the matrix, $H_{tc,tr}$, represent the CSI sequences for each subcarrier.

Secondly, we normalize the matrix, $H_{tc,tr}$, such that each column has a zero mean and unit variance, and we denote the normalized version of the CSI measurement as $Nor_{tc,tr}$.

Thirdly, we calculate the corresponding correlation matrix, portrayed as in Equation (5):

(5) $$C=\left[\begin{array}{cccc} C(1,1) & C(1,2) & \cdots & C(1,N)\\ C(2,1) & C(2,2) & \cdots & C(2,N)\\ \vdots & \vdots & \ddots & \vdots \\ C(N,1) & C(N,2) & \cdots & C(N,N) \end{array}\right],$$

Finally, R-DEHM performs eigendecomposition of the correlation matrix, C, to obtain the eigenvectors, $Ev=(eig_{1},eig_{2},\cdots ,eig_{i})$, and simultaneously structures the principal components,$Pl=(pl_{1},pl_{2},\cdots ,pl_{i})$, which is shown in Equation (6):

(6) $$pl_{i}=Nor_{tc,tr}\times eig_{i},$$

where $eig_{i}$ and $pl_{i}$ are the *i*th eigenvector and *i*th principal component, respectively.

#### 5.2.2. Feature Extraction

**Figure 4:**
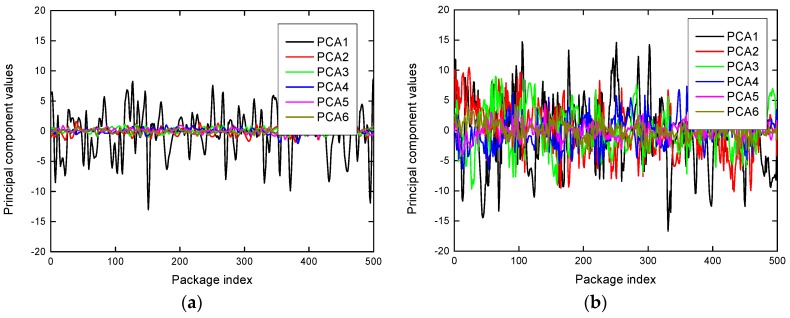
The principal components in two statement environments: (**a**) Top six principal components in the absence environment; (**b**) top six principal components in the presence environment.

**Figure 5:**
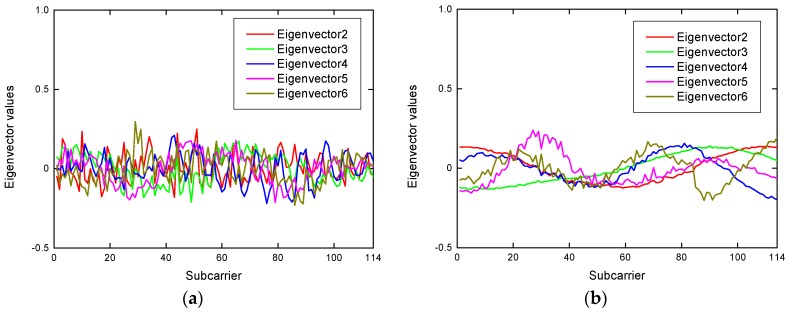
The eigenvectors in two statement environments: (**a**) The remaining eigenvectors in the absence environment; (**b**) the remaining eigenvectors in the presence environment.

To extract the robustness features that are sensitive to the environment and human motion, we analyzed the eigenvectors and the corresponding principal components of the CSI measurements after the PCA-based dimension reduction. To identify the robust features for human motion detection, we analyzed the characteristics of the eigenvectors and the corresponding principal components. Through our experiments, there were two main observations. On the one hand, Figure 4 portrays the principal components of the CSI measurements in the absence scenario and the presence scenario. It can be seen in Figure 4 that the first principal components both have great fluctuations in the two environments. It is impossible to make an adequate distinction between presence and absence for the first principal component. Consequently, the first components are eliminated when extracting detection features in R-DEHM. Further, it can be observed in Figure 4 that there is feeble variation with the remaining principal components in the absence scenario, however, the variation of the principal components is palpable in the presence scenario. On the other hand, Figure 5 compares the eigenvectors’ fluctuation in the absence scenario and the presence scenario. It can be seen in Figure 5 that the remaining adjacent eigenvectors fluctuate gently in the presence scenario and vary in the middle in the absence scenario.

Typically, variance can measure the dispersion degree of data and the first-order difference can reflect the relationship between adjacent data. Thus, we calculated the variance of the principal components, $E^{2}\{pl_{i}\}$, for the *i*th principal component, $pl_{i}$, as shown in Equation (7), and the mean of the first-order difference, $D\{eig_{i}\}$, for the *i*th eigenvector, $eig_{i}$ to expose the corresponding variation characteristics, as shown in Equation (8):

(7) $$E^{2}\{pl_{i}\}=\frac{1}{L}\sum_{i=1}^{L}(pl_{i}-\frac{1}{L}\sum_{j=1}^{L}pl_{j}{)}^{2},$$

(8) $$D\{eig_{i}\}=\frac{1}{N-1}\sum_{l=2}^{N}\left|eig_{i}(l)-eig_{i}(l-1)\right|,$$

where L is the length of the CSI sequential packets, N=114 is the number of subcarriers, and $\left|eig_{i}(l)-eig_{i}(l-1)\right|$ is the difference in coefficients for neighboring subcarriers of the *i*th eigenvector.

According to the experiments in our system, we observed that the ratio, $F\{pl_{i},eig_{i}\}$, of the variance of the principal components to the mean of the first-order difference is more robust as a feature to distinguish whether there is a presence or absence in the vicinity, which is shown in Equation (9):

(9) $$F\{pl_{i},eig_{i}\}=\frac{E^{2}\{pl_{i}\}}{D\{eig_{i}\}},$$

Therefore, to enhance the robustness of the system to human motion detection, R-DEHM focuses on the other principal components except the first principal component, and uses the ratio of variance of the principal components to the first-order differential mean as the feature reflecting the changes of subcarriers.

### 5.3. Human Motion Detection

After extracting the features of the absence and presence environments, R-DEHM realizes human motion detection by classifying different environmental features. If the classification result is presence, R-DEHM will present the result of presence for the current environment, and otherwise, the current environment is presented as the result of absence. To achieve this process, the selection of classifiers is particularly important. The artificial neural network obtains the training model under certain rules through its own training and learning without defining the mapping relationship between the input and output, and after the input value is given, an expected output value can be accurately achieved. As the most widely-used artificial neural network, BPNN is utilized in R-DEHM as the classifier of environmental features to achieve the demarcation line between human presence and absence.

In the construction of BPNN, generally, the number of hidden layers determines the application effect of the classifier. Although the training model may be obtained easily by the multi-hidden layer BPNN, it easily falls into the local minimum. Three-layer BPNN with a single hidden layer can complete arbitrary complex function mapping. Therefore, R-DEHM constructs a simple three-layer BPNN with a hidden layer as the classifier of the environmental feature classification, adopting the momentum gradient descent algorithm with a variable learning rate. The specific classification operation has the following three steps.

First, the data are divided into two categories, i.e., training data and test data.

Then, the features of the training data are selected as the input of the BPNN model. Further, BPNN adjusts the weights and thresholds continuously through repeated learning and training processes, so that the error decreases along the gradient direction. If the error approaches the threshold of 0.01, the learning and training processes will be stopped, thus obtaining the training model of BPNN with a demarcation line for discriminating between different scenarios.

Finally, the features of the test data are classified by using the BPNN training model, and hence human motion detection is realized according to the classification criteria.

### 5.4. Multiple Antennas Fusion Module

With the endorsement of MIMO in modern communication, an increasing number of WiFi devices are equipped with multiple antennas. Generally, not all wireless links are equally sensitive to human movement and the sensitivity varies with the link fade level along with other factors. It is difficult to provide reliable detection in R-DEHM when a specific antenna pair for motion detection is fixed. To avoid this dilemma, we leveraged a simple majority-vote based detection algorithm to ensure the robustness of R-DEHM. Specifically, we executed the human motion detection on each antenna pair, and then we synthesized all results of different antenna pairs by the multiple antennas fusion module to make a final precise human motion detection decision.

### 5.5. Duration Estimation

As a significant parameter of human activity recognition, the duration of human motion is estimated in R-DEHM for the presence CSI data. Considering the continuity of the duration for each presence CSI data, we transformed it into the discreteness of human motion detection. Based on this, a complex duration assessment problem was transformed into a simple human motion detection problem. To be more specific, the duration estimation of the presence CSI data is arranged in three main steps.

Firstly, the entire information of the presence CSI data may be split for discrete analysis. In this case, when the statement of the CSI data is confirmed as a presence in the scenario according to the final human motion detection decision, the pre-processed CSI streams are divided into lots of CSI segments with the same window size, W.

Then, there is a human motion detection process for each CSI segment, including the feature extraction, BPNN, and multi-antenna fusion module. Moreover, the duration of each CSI segment is defined as a window size, W, if the CSI segment is confirmed as a presence in the scenario; otherwise, it should be defined as the value of zero.

Finally, we estimate the duration, T, of the human motion in the scenario, according to the sum of the duration of all CSI segments, which is the statement of a presence in the scenario. Based on this, the duration, T, of the human motion in the scenario can be portrayed as in Equation (10):

(10) $$T=\sum_{s=1}^{num}W,$$

where num represents the total number of CSI segments embodying human motion.

## 6. Implementation and Evaluation

In this section, the prototype implementation and experiment settings of our designed system are presented in detail. Afterwards, we systematically evaluate the performance of the system via real-world experiments.

### 6.1. Prototype Implementation and Experiment Settings

In our experiments, we employed two antennas, a TP-Link wireless router TL-WDR3500 as the transmitter, and an Acer PC equipped with two antennas and Atheros AR9382 NICs was employed as the receiver for pinging packets from the transmitter. According to the antennas’ distribution, a 2×2 MIMO system was constructed by the transmitter and the receiver with four antenna links. In this case, we denoted four antenna links as “Link A”, “Link B”, “Link C”, and “Link D”; the multiple antenna link is denoted as “Multi_Link”, which represents our system. During the detection period, the transmitter sends out beacon messages to the receiver with 1000 packets per second [31]. Then, the receiver gathers these messages along with the CSI and uploads that to the detection server for processing.

**Figure 6:**
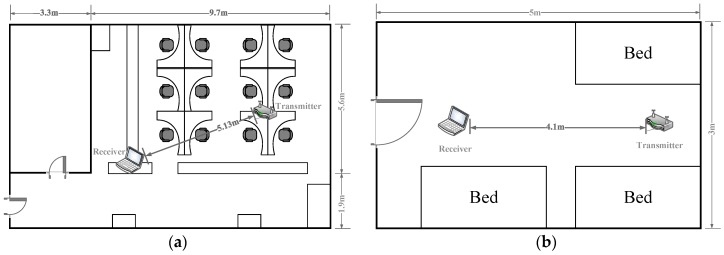
Floor plans for two experiment scenarios: (**a**) Research laboratory; (**b**) graduate dormitory.

To verify the robustness of the system, we conducted experiments under two typical real indoor scenarios, i.e., working area and living quarters. The research laboratory was adopted for the working area and the graduate dormitory for the living quarters, which were set as follows:

(1) **Research Laboratory:** We set up a working area testbed in a 7.5 m×13 m key research laboratory in Hebei University of Engineering, as shown in Figure 6a. The transmitter is placed on the top of the shelters. At the receiver side, the CSI measurements are collected continuously in both the presence and absence environments. The relative position of the transmitter and the receiver is 4.1 m. For the purpose of human motion detection, we generated two data sets covering the entire area of the laboratory, including an absence set and a presence set. The presence set is formed by an individual walking back and forth continuously around the region of interest.
(2) **Graduate Dormitory:** We performed the living quarter experiments in a 3 m×5 m graduate dormitory. In this scenario, the transmitter and receiver are placed in a fixed position as shown in Figure 6b, where the relative position is 5.13 m. We also collected the same number of CSI data over the transmission link, and then the CSI data were uploaded to the system server.

### 6.2. Performance Evaluation

In this subsection, we first present some evaluation metrics for estimation, and then the performances of human motion detection and motion duration estimation are evaluated

#### 6.2.1. Evaluation Metrics

To evaluate the performance of our designed system, we focused on some metrics for estimation, including the true positive, true negative, false positive, false negative, true positive rate, true negative rate, and duration error rate.

- True positive (TP): The TP refers to an event in which a moving human presence is correctly detected.
- True negative (TN): The TN refers to an event in which no human presence is correctly identified.
- False positive (FP): The FP refers to an event in which the system detects the human motion, but there is, in fact, no people moving.
- False negative (FN): The FN refers to an event in which the system detects no human motion, but there is, in fact, people moving.
- True positive rate (TPR): The TPR refers to the probability that the system makes the right judgment in the existence of human motion, interpreting the detection performance in the presence of FN, which can be expressed as in Equation (11):
  (11) $$TPR=\frac{TP}{TP+FN},$$
- True negative rate (TNR): The TNR refers to the probability that the system makes the right judgment in the absence scenario, interpreting the detection performance in the presence of a FP, which can be expressed as in Equation (12):
  (12) $$TNR=\frac{TN}{TN+FP},$$
- Duration error rate (DER): The DER is the ratio of absolute error caused by measurement to the total time. Specifically, the formula can be expressed as in Equation (13):
  (13) $$DER=\frac{\left|Mt-Rt\right|}{St},$$
  where Mt and Rt represent the measured duration and the real duration of human motion, respectively, and St is the total time of the sample.

#### 6.2.2. Performance Evaluation of Human Motion Detection

**Figure 7:**
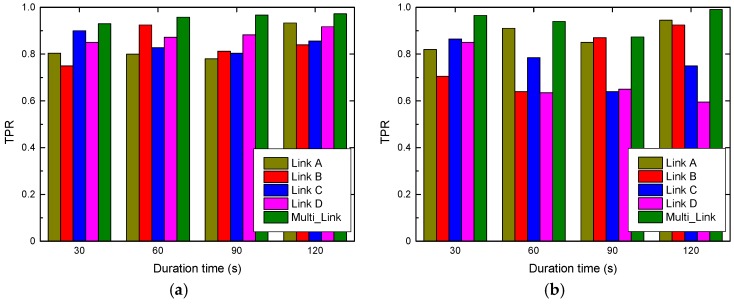
Impact of multiple antenna links in two testbeds: (**a**) True positive rate in a graduate dormitory; (**b**) true positive rate in a research laboratory.

This subsection presents the performance of our designed system in terms of human motion detection. Figure 7 shows the comparison of TPR among different antenna link cases in two diverse scenarios, considering four cases of human motion duration. It is evident that R-DEHM, i.e., Multi_Link, outperforms the single antenna cases in terms of the TP rate, with an improvement of approximately 16.96%. To be more specific, the average TPR by a single antenna link is 81.21% and the average TPR by the multiple antenna links is 94.98%. This is because a simple majority-vote based detection algorithm is leveraged in R-DEHM. Based on this, our multiple antennas fusion scheme achieves an excellent performance, ensuring the stability of human motion detection and the validity of duration estimation of human motion.

Besides, the human motion detection results of all single antennas are unstable, and bad results do not always appear on the same single antenna, such as Link B at 30 s detecting a TPR of 75% and Link A at 90 s detecting a TPR of 78%, as shown in Figure 7a. This is because factors, such as the communication quality between transceivers and the moving position of the target human, have different effects on each link in the process of data acquisition. Furthermore, this affect also occurs in different time periods for the same antenna link. It can be observed in Figure 7 that the fluctuation of TPR in the graduate dormitory is smaller than that in the research laboratory. This is because the distance between the transceivers is relatively small in the narrow dormitory space, and the range of activity of the target human is also relatively close to the line of sight of the transceivers. Thus, wireless signals in graduate dormitory more easily capture activity information.

Table 1 shows the comparison of TNR among different antenna link cases in two diverse scenarios. R-DEHM detects human motion, leveraging a simple majority-vote based detection algorithm with an average TNR almost close to 100%. Moreover, we observed that the TNR of all simple antenna link cases is less than 95% except for Link B in the graduate dormitory. After checking the trace, we found that the false alarms are almost always caused by a sudden increase in noise levels. We also found that the TNR in the research laboratory is inferior to the TNR in the graduate dormitory. This is because the space distribution of the laboratory is complex, and hence the result are easily affected by the multi-path.

#### 6.2.3. Performance Evaluation of Motion Duration Estimation

**Figure 8:**
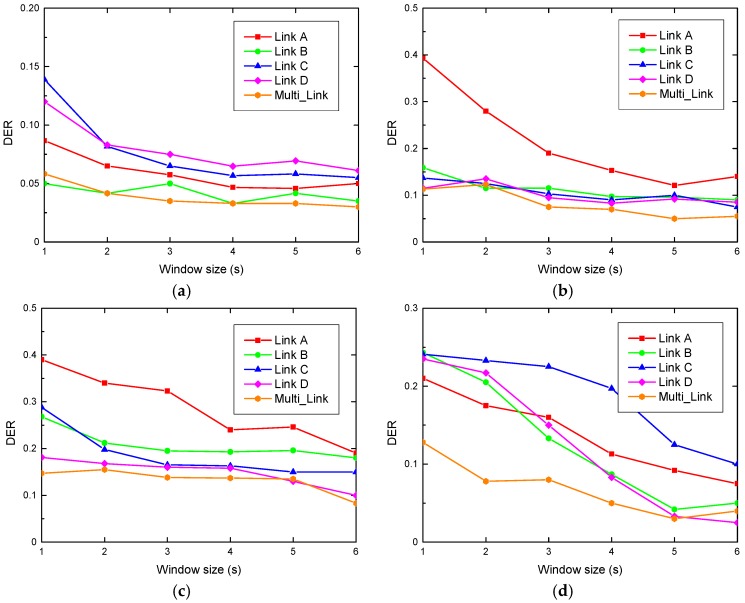
The DER in the dormitory: (**a**) DER with 30 s; (**b**) DER with 60 s; (**c**) DER with 90 s; (**d**) DER with 120 s.

**Figure 9:**
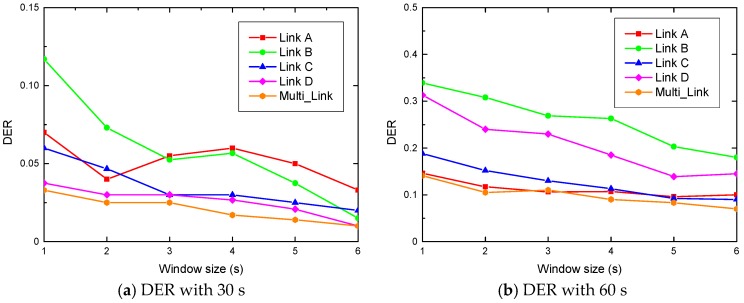

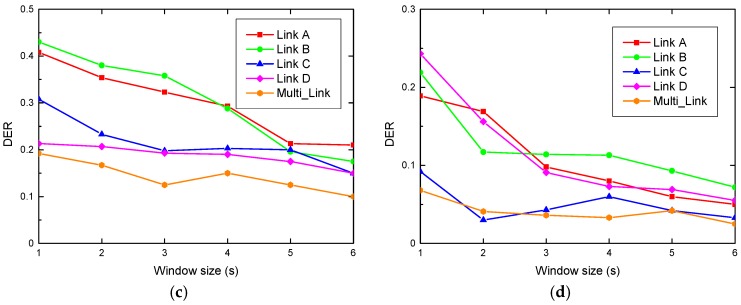
The DER in laboratory: (**a**) DER with 30 s; (**b**) DER with 60 s; (**c**) DER with 90 s; (**d**) DER with 120 s.

This subsection presents the performance of our designed system in terms of motion duration estimation. Figure 8 and Figure 9 show the DER of duration estimation for human motion by four cases of human motion duration in a graduate dormitory and research laboratory, respectively. We observed that the DER using multiple antenna links is lower than that of the single antenna link. This is because the position of the target human changes during the course of their activities, which leads to different changes of each antenna link. Therefore, it is almost impossible to express the information of the activity completion by using a single antenna to estimate human motion duration. On the contrary, when leveraging the simple majority-vote based detection algorithm, the information of each antenna can be synthesized to estimate the duration of human motion more completely. Besides, we noticed that R-DEHM estimates the human motion duration by leveraging a simple majority-vote based detection algorithm with an average DER less than 10%.

We also observed that the DE rate decreases with the increase of the window size. This is because the variance of a CSI segment changes more obviously as the window size is enlarged. In this case, when the variance of a CSI segment is much smaller than the human motion duration, the system is more sensitive to the moving information. Furthermore, it can be seen that we cannot identify which single antenna link case has the worst or best performance of DER. This is because there is no stable sensitivity for all single wireless links to detect human motion under any situation. Furthermore, it is evident that the mean fluctuation of DER in the graduate dormitory is smaller than that in the research laboratory. This is because of the difference in the space distribution, which is the same reason for the TPR trend differences. There is less multi-path impairment when the smaller enclosed scenario is adopted for the experiment.

Table 2 shows the average DER with different antenna links in the graduate dormitory and research laboratory. It can be seen that the average DER rates with a single antenna link in the graduate dormitory and research laboratory are 13.7% and 14.25%, respectively. Moreover, the average DER with multiple antenna links in the graduate dormitory and research laboratory are 8% and 7.61%, respectively. Since the simple majority-vote based detection algorithm was employed in our designed system, the improvements of DER in the graduate dormitory and research laboratory are 41.61% and 46.6%, respectively. We observed that the DER improvement of the research laboratory is larger than that of the graduate dormitory. This implies that the scheme of multiple antennas fusion is of great significance to R-DEHM and it works especially well in complex environments. Besides, Table 2 shows that the average DER of Link A in the dormitory and Link B in the laboratory are relatively high, with both exceeding 15%. Thus, it proves once again that it is impossible to fix a specific antenna link for duration estimation of human motion.

## 7. Conclusions

With the development of wireless technology, wireless human behavior recognition has attracted much attention, but there is lack of consideration of the duration of human motion. In this paper, we presented a channel statement information (CSI)-based device-free system for robust duration estimation of human motion with commodity WiFi devices, and the design and implementation of the system were presented in detail. To the best of our knowledge, this is the first system that utilizes the CSI from the physical layer for duration estimation of human motion. A fresh perspective was proposed to transform the complex and continuous duration estimation problem into a simple and discrete human motion detection problem. We prototyped the system and evaluated it in two real environments, containing the effectiveness of human motion detection and duration estimation. The evaluation results presented that the average true positive rate (TPR) and true negative rate (TNR) of human motion detection with multiple antenna links are larger than 94% and close to 100%, respectively. Simultaneously, the average duration error rate (DER) of duration estimation of human motion with multiple antenna links in a graduation dormitory and research laboratory are 8% and 7.61%, respectively, which provides evidence of the effectiveness of R-DEHM. In the near future, more environmental factors should be considered to optimize our methods for more accurate estimation. Furthermore, we will strive to achieve the motion duration estimation of multiple humans in our future work.

## Figures and Tables

**Table 1:** TNR among different antenna link cases in different scenarios.

	Antenna	Link A	Link B	Link C	Link D	Multi_Link
Scenario						
Dormitory		0.89	0.96	0.90	0.93	1.00
Laboratory		0.90	0.94	0.90	0.80	0.99

**Table 2:** The average DER with different antenna links in two scenarios.

	Antenna	Link A	Link B	Link C	Link D	Multi_Link
Scenario						
Dormitory		17.43%	12.2%	13.84%	11.33%	8%
Laboratory		14.23%	18.62%	10.7%	13.43%	7.61%
